# Cranial ultrasound findings in preterm germinal matrix haemorrhage, sequelae and outcome

**DOI:** 10.1038/s41390-020-0780-2

**Published:** 2020-03-26

**Authors:** Alessandro Parodi, Paul Govaert, Sandra Horsch, Marìa Carmen Bravo, Luca A. Ramenghi, eurUS.brain group, Thais Agut, Ana Alarcon, Roberta Arena, Marco Bartocci, Mayka Bravo, Fernando Cabañas, Nuria Carreras, Olivier Claris, Jeroen Dudink, Monica Fumagalli, Paul Govaert, Sandra Horsch, Alessandro Parodi, Adelina Pellicer, Luca A. Ramenghi, Charles C. Roehr, Sylke Steggerda, Eva Valverde

**Affiliations:** 1 IRCCS, Istituto Giannina Gaslini, DINOGMI Department University of Genoa, Via Gaslini 5, 16148 Genoa, Italy; 2 Department of Neonatology, Erasmus Medical Center University, Sophia Children’s Hospital, Rotterdam, The Netherlands; 3 Department of Neonatology, ZNA Middelheim, Antwerp, Belgium; 4 Department of Rehabilitation and Physical Therapy, Gent University Hospital, Gent, Belgium; 5 Department of Neonatology, Helios Klinikum Berlin Buch, Berlin, Germany; 6 Department of Neonatology, La Paz University Hospital, Madrid, Spain; 7 Department of Neonatology, Institut de Recerca Pediàtrica, Hospital Sant Joan de Déu, Barcelona, Spain; 8 Catholic University of the Sacred Heart, A. Gemelli Hospital, Rome, Italy; 9 Department of Women’s and Children’s Health, Karolinska University Hospital, Karolinska Insitute, Stockholm, Sweden; 10 Department of Neonatology, Quironsalud Madrid University Hospital and Biomedical Research Foundation, La Paz University Hospital Madrid, Madrid, Spain; 11 Service de néonatologie et de réanimation néonatale, Hospices Civils de Lyon, Université Claude Bernard Lyon, Villeurbanne, France; 12 UMCU-Wilhelmina Children’s Hospital, Lundlaan 6, 3584 EA Utrecht, The Netherlands; 13 Department of Clinical Sciences and Community Health, University of Milan, Milan, Italy; 14 Fondazione IRCCS Ca’ Granda Ospedale Maggiore Policlinico NICU, Milan, Italy; 15 Department Clinical Science Intervention and Technology (CLINTEC), Karolinska Institutet, Stockholm, Sweden; 16 Department of Paediatrics, Medical Sciences Division, Newborn Services, University of Oxford, Oxford, United Kingdom; 17 Department of Neonatology, Leiden University Medical Center, Leiden, The Netherlands

## Abstract

Germinal matrix-intraventricular haemorrhage (GMH-IVH), periventricular haemorrhagic infarction (PHI) and its complication, post-haemorrhagic ventricular dilatation (PHVD), are still common neonatal morbidities in preterm infants that are highly associated with adverse neurodevelopmental outcome. Typical cranial ultrasound (CUS) findings of GMH-IVH, PHI and PHVD, their anatomical substrates and underlying mechanisms are discussed in this paper. Furthermore, we propose a detailed descriptive classification of GMH-IVH and PHI that may improve quality of CUS reporting and prediction of outcome in infants suffering from GMH-IVH/PHI.

## Introduction

### Epidemiology

Despite improvement in the care of preterm infants, germinal matrix-intraventricular haemorrhage (GMH-IVH) and parenchymal haemorrhagic infarction (PHI) remain feared complications in this vulnerable population. The overall incidence ranges between 20 and 25% among very low birth weight (VLBW) infants.^1^ The risk of GMH-IVH increases with decreasing gestational age:^2–4^ in surviving infants born at 24 weeks of gestation, the incidence of the most severe lesions (i.e. grade III GMH-IVH and PHI; the rationale for grading is explained below) ranges between 10 and 25%, while in surviving infants born beyond 28 weeks, such severe injury is diagnosed in <5% of cases.^2–4^ GMH-IVH is rarely observed beyond 32 weeks gestation:^3^ in such late-onset cases, it is an epiphenomenon of other diseases like venous thrombosis.^5–7^

### Pathogenesis

**Fig. 1:**
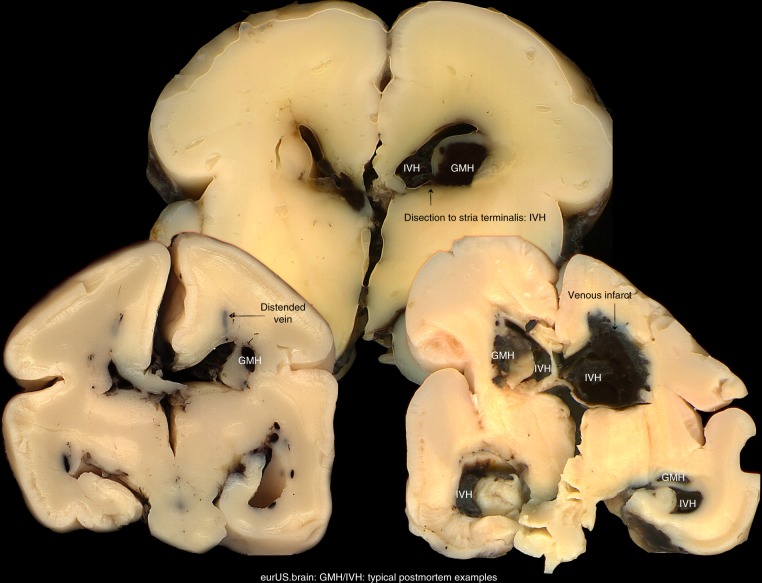
GMH/IVH: typical postmortem examples.

The pathogenesis of GMH-IVH and PHI is multifactorial and complex. Gestational age is the most important single independent risk factor. The germinal matrix reaches its maximum volume around 25 weeks gestation and subsequently withers. A residual mass persists until ~36 weeks gestational age.^8–10^ A venous origin of GMH-IVH has been demonstrated by postmortem studies^11,12^ (Fig. 1). Intrinsic fragility of germinal matrix microvasculature due to immaturity of the vessel wall, fluctuations in cerebral blood flow and the lack of autoregulation seem to represent important contributing factors.^13–16^ Fluctuations in venous pressure, variations in venous anatomy and genetic factors are also part of this complex interplay.^17–20^ Several clinical conditions have been associated with GMH-IVH: perinatal hypoxic–ischaemia, inflammation, cardiovascular instability, severe respiratory disease, pneumothorax, inotropic drug use and many more.^21^ All of them induce fluctuations in CBF, which in turn increase the risk of bleeding from fragile venules. The only single independent factor that has been proven to decrease the risk of GMH-IVH and improve long-term outcome is lung maturation by antenatal glucocorticosteroid treatment.^22–24^ Postnatal indomethacin has been shown to reduce the rate of severe IVH, particularly in male infants,^25^ but did not improve neurodevelopmental or sensory long-term outcome.^26^ Data on the preventive effect of delayed cord clamping are still conflicting, especially when the intrinsic risk of low gestational age is considered.^27,28^

### Time of onset

Postnatal GMH-IVH and PHI occur nearly exclusively during the first week of life. In at least 50% of affected infants the onset of GMH-IVH is on the first day of life, and by 72 h, ~90% of the lesions are identified. Progression to higher grades occurs rapidly, within 1–3 days.^29–33^ It is striking that premature infants are relatively immune to haemorrhage after the first week of life, irrespective of gestational age. This reduced vulnerability might be related to an increase in blood and tissue oxygen concentration after birth, suppressing vascular endothelial growth factor and angiopoietin-2 levels: a shutdown in angiogenesis after birth would induce maturation of vessels making them resistant to rupture.^21,34^

Foetal intracranial haemorrhage is not uncommon, with an estimated incidence of 0.5–0.9/1000 pregnancies. Antenatal GMH-IVH is the most common type of foetal intracranial haemorrhage.^35–37^ A cranial ultrasound (CUS) on admission allows pre-existing antenatal brain injury to be identified. If antenatal GMH-IVH occurred well before birth, residual findings (ventricular dilatation, intraventricular clots and strands, parenchymal defects) may be subtle (see other relevant paper in this issue). PHI with an atypical time of onset (antenatal or after 96 postnatal hours when unrelated to a clinical deterioration) has been associated with the presence of thrombophilic disorders, especially factor V Leiden.^17^

### Clinical presentation in preterm infants

Most instances of GMH-IVH/PHI are clinically silent and detected during routine CUS. Some infants manifest with subtle changes in the level of consciousness, limb movement, tone, and eye movement in the hours to days after the IVH. GMH-IVH and PHI can be accompanied by various degrees of cardiorespiratory instability and anaemia. With extensive haemorrhage, a catastrophic deterioration occurs with stupor, “decerebrate” posturing, generalized tonic seizures and hypotonia.^1^

### Outcome of grade I–III GMH-IVH

Infants with grade III GMH-IVH carry a significantly increased risk of neurodevelopmental disability, especially when GMH-IVH is complicated by post-haemorrhagic ventricular dilatation (PHVD) that needs surgical intervention. Cerebral palsy rates in infants with IVH III range between 7 and 63%^38–40^ and reflect the heterogeneity of this collective. Infants that suffer low-grade (i.e. grade I or II) GMH-IVH are clearly at much lower risk of developmental disabilities compared to infants with grade III GMH-IVH or PHI. Therefore, it has been customary to inform parents that the finding of an uncomplicated low-grade GMH-IVH has no relevant impact on later neurodevelopment and academic achievement. Recent data, though, suggest that this may not be entirely true.^40,41^ It has been shown that low-grade GMH-IVH is followed by microstructural impairment in periventricular and subcortical white matter.^42^ Size, number and location of these minor lesions might be of great importance in infants born at the lowest gestational ages, although robust data are lacking. Matrix injury even after an uncomplicated GMH-IVH results in a relevant loss of glial precursor cells, leading to impaired myelination and cortical development.^43,44^ GMH-IVH further triggers inflammation in adjacent white matter through activated microglia, passage of red blood cells and red blood cell degradation: the resulting perilesional tissue injury may be secondary to free radical release and the presence of free iron.^45–48^ The outcome of grade I and II GMH-IVH needs to be prospectively studied in relation to the exact description of size and location in different parts of the matrix protomap of telencephalic development. Similar relevant side effects of intraventricular blood products on the cerebellar external granular layer, after passage into the cerebellar subarachnoid spaces, are not discussed here.

### Complications and their outcome

#### Parenchymal haemorrhagic infarction

PHI complicates GMH-IVH in ~15% of cases.^49,50^ GMH-IVH of all grades can be complicated by PHI, but the higher the grade of GMH-IVH, the more likely PHI is to occur.^1,51^ PHI is caused by venous obstruction induced by GMH-IVH. Venous congestion leads to ischaemia and to secondary haemorrhagic infarction. High intraventricular pressure due to a large haemorrhage may additionally affect flow through the subependymal veins, increasing infarct size.^52^ Cerebral palsy and severe cognitive impairment are common in infants who suffered from PHI.^38–40^ Prognosis is highly dependent on location and extent.^53–56^ Classifying PHI into venous subtypes helps to predict outcome and counsel parents in this difficult situation,^55^ and this should be expanded in relation to specific behavioural or cognitive sequelae. Mortality in infants with extensive PHI is high, especially when it occurs bilaterally. In many countries, redirection of care and end of life decisions are considered in infants with bilateral PHI. Although robust data on this are lacking, it is certain that redirection of care contributes significantly to reported mortality rates.^57,58^

#### Post-haemorrhagic ventricular dilatation

The term PHVD refers to dilatation of the ventricles subsequent to GMH-IVH. Approximately 25% of infants with GMH-IVH develop progressive PHVD.^59^ The risk of PHVD is higher following severe GMH-IVH (i.e. grade III GMH-IVH or PHI).^60^ The vast majority of instances of progressive PHVD (80%) follow IVH III, often in combination with a PHI. Most often it follows obstruction of liquor pathways around the cerebellum. While in most cases PHVD eventually resolves (~40% spontaneously and another 15% after non-surgical treatment), around 35% of infants with progressive PHVD require surgical treatment, while 10% die.^59^ Despite decades of extensive research, treatment of PHVD remains challenging.^61^ Several options were investigated: lumbar or ventricular tapping, cerebrospinal fluid (CSF) drainage and fibrinolytic treatment, surgical insertion of an external drain, a subcutaneous reservoir and permanent ventriculo-peritoneal shunting. The key problem is to balance between the adverse effects of PHVD on the immature brain and the risk of complications of interventions (e.g. infection related to CSF tapping, secondary bleeding after fibrinolytic treatment, development of a trapped fourth ventricle).^62^ PHVD is strongly associated with neurodevelopmental impairment, particularly in infants with persistent PHVD that requires surgical intervention and if PHVD is combined with PHI.^39^ However, more recent studies revealed a better outcome than reported earlier.^38,63^ This might partly reflect the heterogeneity of management strategies among centres. A recent multicenter study showed that early treatment of PHVD, based on ventricular measurements, is associated with favourable neurodevelopmental outcomes, even when a permanent shunt is eventually needed.^64^

## The role of ultrasound in diagnosing GMH-IVH and its complications

### Screening for GMH-IVH in the NICU

Preterm infants are often unstable during the first days of life, when GMH-IVH typically presents. CUS allows prompt diagnosis of GMH-IVH as well as assessment of the evolution.^65^ In the most critical phase, CUS should be as “quick and gentle” as possible, in order to minimize stress for fragile neonates. As the incidence of GMH-IVH is closely related to gestational age at birth,^66^ it is reasonable to recommend the following schedule: in preterm infants with a gestational age below 28 weeks or 1000 g, serial CUS should be performed on days 1, 3, 7, 14, 21, 28, and then every other week until term-equivalent age. In stable preterm infants with a gestational age above 28 weeks, the frequency of serial CUS can be limited to days 1, 3, 7, 14,d 28, at 6 weeks and at term-equivalent age. Additional targeted examinations may be warranted in case of clinically or ultrasonographically suspected bleeding with uncertain findings.^67^ CUS examinations beyond the first week of life allow detection of PHVD as well as of uncommon occurrence of late-onset GMH-IVH.

### Grading of GMH-IVH

**Fig. 2:**
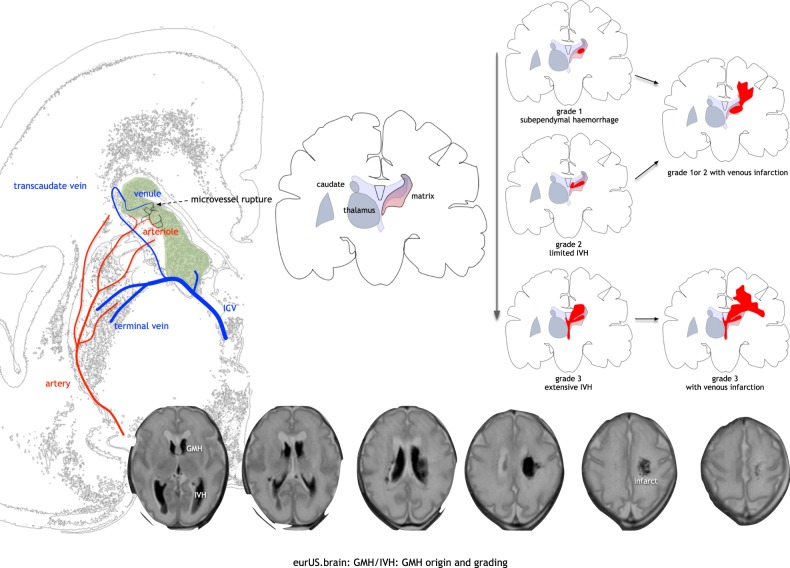
GMH/IVH: origin and grading. GMH starts in a venule that drains into lateral subependymal collector veins; it extends into white matter by virtue of venous compression and infarction; bottom row: T2-weighted MRI of GMH with limited IVH and limited venous infarct.

An early grading system for GMH-IVH was proposed by Papile et al.^68^ in 1978, based on severity assessed by computed tomography. Despite several ultrasound classifications subsequently published,^69,70^ the Papile classification has been widely used for decades by clinicians and researchers.^71^ It suggested that progression from a grade I to a grade IV haemorrhage represents a continuum, as follows: subependymal bleeding limited to germinal matrix (grade I), intraventricular haemorrhage extending into normal sized ventricles and typically filling <50% of the ventricular lumen (grade II), intraventricular haemorrhage extending into dilated ventricles (grade III), intraventricular haemorrhage with parenchymal extension (grade IV). However, the formerly called “grade IV” represents periventricular haemorrhagic venous infarction (PHI), rather than parenchymal extension by rupture into the parenchyma of the initial GMH-IVH—although this event very rarely does occur^52,72^ (Fig. 2). As PHI can be associated with any grade of GMH-IVH, a classification into three grades with separate notation for the presence of PHI, like the Volpe classification, is strongly suggested, both in clinical practice and in research context.^1^ Nevertheless, subependymal GMH and very limited IVH (intraventricular blood <10% of the ventricular lumen) were lumped under grade I in Volpe classification, making the distinction between these two entities irrelevant.^1^ In light of the above-mentioned studies showing the potential detrimental effects of intraventricular blood on the developing brain, subependymal GMH and limited IVH were separated into two grades in the new classification system we propose in the present paper (Table 1). Furthermore, despite some studies showing that germinal matrix injury results in a relevant loss of glial precursors, the impact of subependymal GMH on neurodevelopment in relation to its extent and location is still unknown:^43,44^ it may therefore become a necessity to further detail descriptions of the extent and location of GMH in the future, although this new classification system needs to be validated in a large cohort of patients before it can be recommended in routine clinical practice.

**Table 1:** GMH/IVH description.

GMH	IVH	Venous infarction (PHI)	
Size			
Largest diameter < 1 cm	Limited	Medullary	Entire frontal white matter up to the atrium
Largest diameter ≥ 1 cm	extensive		Above caudate head only
Location near caudo-thalamic groove			
Above caudate head in front of the foramen of Monro			Thalamostriate (terminal) vein area complete (Monro to atrium) partial
Above caudate nucleus behind the foramen of Monro			Inferior ventricle vein area
Other location			
Along temporal horn			Other Lateral atrial vein Direct lateral vein
Other		Midline	Septal
			Callosal
		Striatal	Superior (thalamostriate vein territory)
			Inferior (basal vein territory)

### Ultrasonographic diagnosis of GMH-IVH

**Fig. 3:**
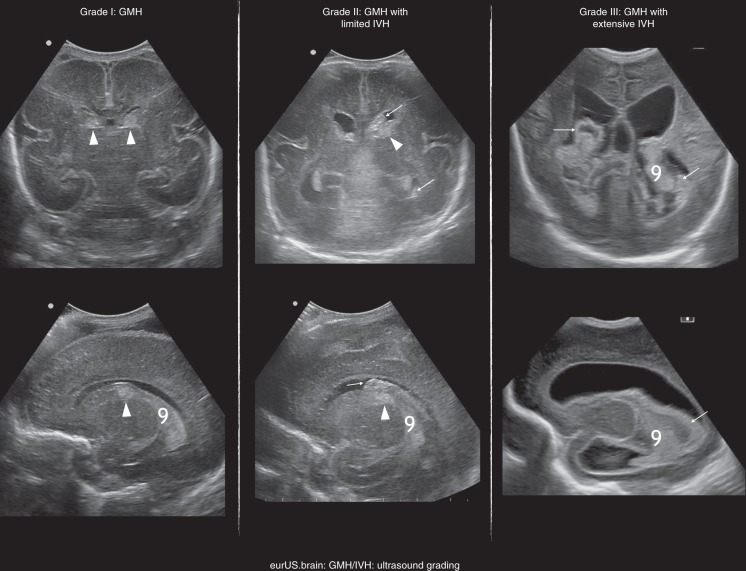
GMH/IVH: ultrasound grading. CUS grading of GMH/IVH; arrowheads point to GMH, arrows to the presence of clot in the ventricle cavity; asterisk is choroid plexus.

When bleeding is limited to the germinal matrix (GMH), the typical CUS finding is a subependymal hyperechoic globular thickening detected during the first week of life, which usually remains visible for a few weeks (Fig. 3). As in some cases, it may be difficult to distinguish a small subependymal GMH from adjacent hyperechoic choroid plexus, both coronal and parasagittal scans should be carefully examined before a diagnosis of GMH is made.^69,73,74^ The most anterior portion of choroid plexus is thin and fills the foramen of Monro at the level of the caudo-thalamic groove; plexus thickens posteriorly, often showing pulsations, is not visible in the frontal and occipital horns of the lateral ventricle and produces a near symmetrical picture in coronal sections.^75^ Asymmetric hyperechoic thickening at the caudo-thalamic groove (the most common location for GMH) in coronal planes occurring in the first postnatal days strongly suggests unilateral GMH. Of course, GMH may also occur bilaterally.^69,72^ Conversely, an echogenicity developing at the groove in the late neonatal period should suggest hyperechoic germinolysis rather than late GMH.^76,77^ Postmortem studies confirmed that the majority of GMHs develop in the caudo-thalamic region, although at postmortem GMH has been described also in occipital and temporal horns: these regions should be carefully examined during CUS.^72,78^ Neuroimaging studies have demonstrated that diagnostic accuracy of CUS for minor forms of GMH-IVH is suboptimal when compared to the MR-SWI sequence (magnetic resonance-susceptibility weighted imaging), which is considered the most sensitive technique for detection of subtle haemorrhage.^79^

The ependymal layer surrounding the germinal matrix may rupture, allowing extension into the ventricular lumen (IVH), typically at the stria terminalis. IVH can be “limited” or “extensive”: in practice “extensive” (grade III according to Papile and Volpe classification) is reserved for haemorrhage that leads to acute ventricle distension by clot and not by CSF, but this remains arbitrary, as measurement of clot volume is not possible with ultrasound. The presence of IVH is usually suggested by intraventricular hyperechoic clot located anteriorly to the foramina of Monro, above the caudo-thalamic groove or in the occipital horn. In the latter case, insonation through the posterior or even mastoid fontanelle allows better visualization of the clot.^69,75^ However, identification of a minimal amount of intraventricular blood (i.e. the distinction between “pure” subependymal GMH and a limited IVH) remains challenging. In this context, indirect signs can corroborate a diagnosis of IVH:^80^ hyperechoic ependymal changes appear from 2 to 4 weeks after IVH, while clots from supratentorial origin can be detected in the fourth ventricle and around the cerebellar surface by insonating through the mastoid fontanelle. Ventricular dilatation by large clot (grade III according to Papile and to Volpe classification, or extensive IVH) should be distinguished from PHVD, which takes place after days or weeks due to distal obstruction of CSF circulation and perhaps later to impaired resorption.^69^ Ultrasonographic characteristics of clot changes over time should be always kept in mind in order to consider whether IVH could be of antenatal origin when subacute characteristics of the clot are observed soon after birth, or to suspect an IVH already in its earliest, hyperacute phase. Hyperechogenicity in the acute stage is due to fibrin formation at the end of the clotting cascade and is the main feature of the clot between 4 and 6 h and 3 days after the bleeding.^81^ In the earliest, hyperacute phase, fresh IVH may remain hypo- or isoechoic, and motion of particulate CSF can sometimes be visible within the ventricles (video particulate CSF with motion). In the subacute phase, after an initial retraction, clot is characterized by progressive hypoechoic change in the central portion and by hyperechoic margins. Intraventricular fibrin strands around the clot can be observed (Fig. 3). In some cases, intraventricular clot fragments remain detectable for months (chronic phase).^69,74,75^ The antenatal origin of an IVH is often suggested by the detection of a subacute clot or clot remnants on the first day of life.

**Fig. 4:**
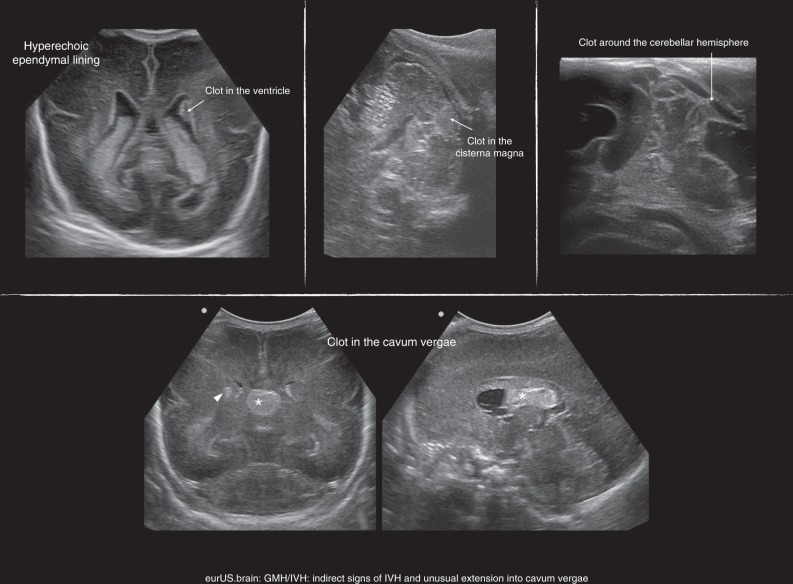
GMH/IVH: indirect signs of IVH and unusual extension into cavum vergae. Arrowhead points to GMH; asterisk represents clot in midline cavity.

Besides cerebellar haemorrhage, which is often detected in patients with GMH-IVH, an uncommon type of bleeding that can be associated with GMH-IVH is septal haemorrhage. The typical finding is the presence of a clot in the cavum septi pellucidi and/or in the cavum vergae (Fig. 4). Septal haemorrhage may derive from septal veins or from extension of IVH following the rupture of one septal leaflet.^82^ Plexus haemorrhage has been described associated to GMH-IVH in neuropathological studies of VLBW infants.^72,83^ However, discriminating plexus haemorrhage from GMH-IVH is difficult for the sonographer in the acute stage.

### Ultrasonographic diagnosis of complications of GMH-IVH

#### Periventricular haemorrhagic infarction

**Fig. 5:**
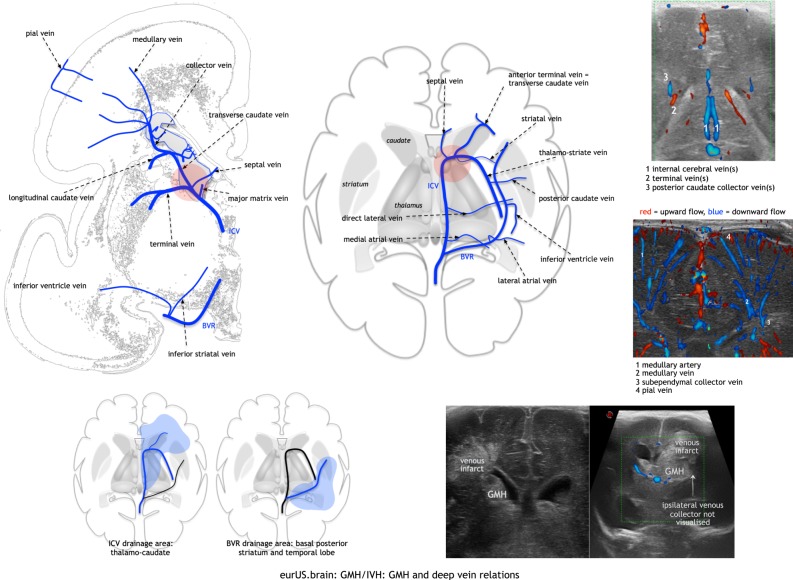
GMH/IVH: GMH and deep vein relations. Deep venous anatomy and some Doppler examples; in red circles the typical location of GMH near the caudo-thalamic groove; initially the GMH is often separate from a resulting venous infarct; the two may merge, and extensive lesions can be associated with absent terminal vein drainage.

Periventricular haemorrhagic infarction (PHI), also referred to as parenchymal haemorrhagic infarction or periventricular venous infarction or intraparenchymal lesion, can complicate each grade of GMH-IVH and seems to occur a few hours up to a few days after the initial bleeding.^84^ Postmortem and Doppler studies strongly suggest that this lesion is due to infarction following venous obstruction and congestion.^52,85–87^ It is also likely that arteriolar hypoperfusion secondary to venous obstruction contributes to parenchymal injury (ref.;^88^ Fig. 5).

**Fig. 6:**
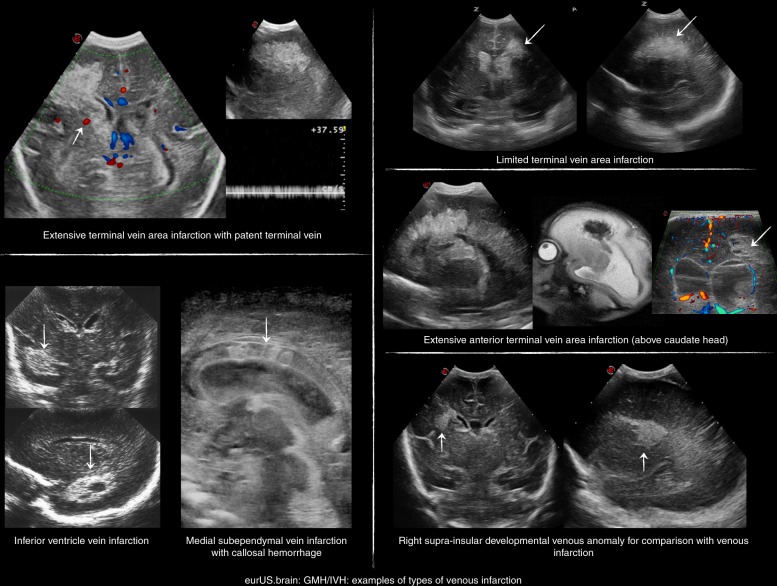
GMH/IVH: examples of types of venous infarction. Examples of different types of venous infarction: thalamostriate (terminal vein), anterior terminal vein (caudate), inferior ventricle vein, medial subependymal (midline) veins, compared with the image of typical posterior frontal developmental venous anomaly (DVA).

The characteristic ultrasonographic appearance of PHI is a triangular, “fan-shaped” echodensity in periventricular white matter, ipsilateral to GMH-IVH^51,52^ (Fig. 6). In the earliest phase, the lesion appears unattached to the ventricle wall; it may subsequently grow, touch the ventricle wall and eventually merge into a single, large hyperechoic lesion together with the initial GMH-IVH. The parenchymal hyperechoic area tends to decrease after few days: this is believed to reflect the resolution of venous congestion in the area surrounding the infarction, which may lead the sonographer to overestimate the extent of PHI during the acute phase.^75^ PHI often remains separate from the initial GMH, appearing as a small hyperechoic lesion in ipsilateral periventricular white matter. Multiple minute PHIs along the course of the medullary veins can also be observed. We speculate that such minor PHIs may result from partial obstruction but not occlusion due to compression of a subependymal collector vein by the GMH. The risk of developing PHI following a subependymal GMH might be related to the location of the GMH itself, especially in association with a peculiar venous anatomy prone to congestion, for example, the presence of acute venous angles.

**Fig. 7:**
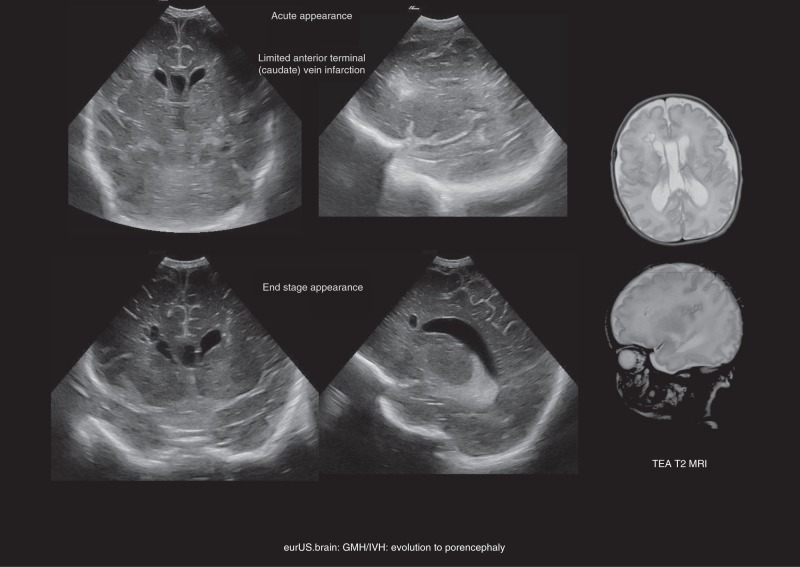
GMH/IVH: evolution to porencephaly.

PHI usually evolves into cavitation within periventricular white matter. As most PHIs develop adjacent to the ventricular wall, porencephaly resulting from the cavitation is common after 1 or 2 months^89–91^ (Fig. 7). Regardless of the evolution into porencephaly, a cavitation resulting from PHI is usually single, asymmetric and persistent. Conversely, cysts of periventricular leukomalacia typically show a symmetrical, mainly posterior distribution and tend to disappear within few weeks, insomuch that they are often undetectable at term-equivalent age.^1,92^

**Fig. 8:**
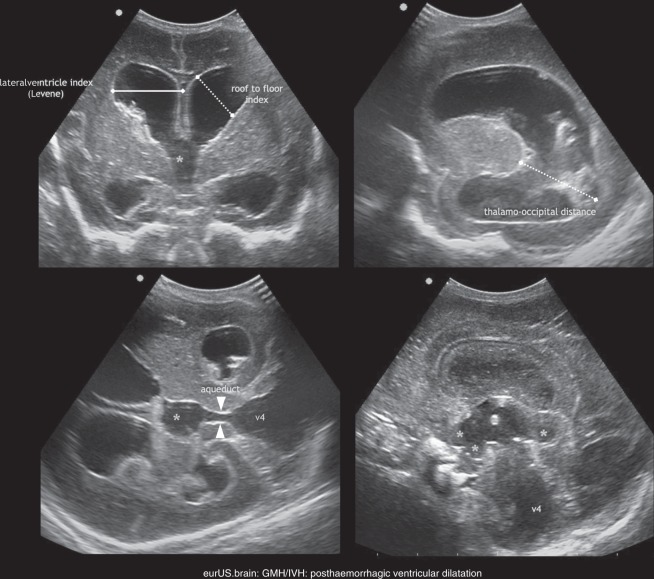
GMH/IVH: post-haemorrhagic ventricular dilatation. Measurements and inspection of dilated ventricles following extensive IVH (* third ventricle with extended protrusions).

Size and location of PHI depend on which vein is obstructed. In some cases, more veins are involved, leading to extensive unilateral or rarely bilateral PHI.^54,55^ Because a diagnosis of PHI carries prognostic implications and may raise ethical issues in severe cases,^53,58^ we propose that classification of PHI should be adopted in routine clinical practice. Dudink et al.^55^ classified PHI into venous subtypes and showed how their classification correlates with motor outcome. Advanced MRI techniques like DTI (diffusion tensor imaging) add useful information for prediction of outcome.^93^ Nevertheless, access to MRI is difficult in low-income countries, limited by obvious logistic obstacles in the acute or early subacute phase of PHI due to clinical instability of the infant. In this context, recognizing the venous subtype of PHI rather than labelling the lesion as an unspecified “PHI” (or “grade 4”) can help the clinician to predict neurological outcome, allowing the start of a targeted rehabilitation programme at an early stage, and may enrich the quality of counselling (see Table 1, Figs. 7 and 8).

#### Post-haemorrhagic ventricular dilatation

PHVD is caused by an imbalance between production and circulation and/or resorption of CSF. It usually develops a few days to a few weeks after the initial IVH, although exceptionally rare cases developing after term-equivalent age have been reported.^94,95^ Although PHVD is more frequently observed after grade III GMH-IVH and PHI, it may complicate each grade (provided that bleeding has extended into the ventricular lumen).^59^ For this reason, serial ultrasound scans are warranted following GMH-IVH at least until term-equivalent age (Fig. 8). Obstruction to CSF circulation at various levels by clots or by fibrin debris initially contribute to ventricular enlargement. The patient can develop various types of ventricular dilatation according to the location of the obstruction(s): unilateral PHVD following unilateral obstruction at the foramen of Monro, supratentorial (triventricular) PHVD following aqueduct obstruction, complete internal (tetraventricular) hydrocephalus following obstruction of the fourth ventricle outlets (foramina of Luschka and Magendi), combined internal and external hydrocephalus (also referred to as communicating hydrocephalus) following impairment of CSF reasorption in the peritentorial arachnoid spaces. In some cases, the fourth ventricle is isolated from CSF circulation by combined obstruction of aqueduct and the fourth ventricle outlets.^59,95,96^ One relevant advantage of CUS is the possibility to perform repeated scans in order to timely detect PHVD and to follow its evolution before and after treatment. Initial dilatation is often transient with a stable phase followed by regression of dilatation within days or a few weeks.^59,97^ Progression of PHVD can be described with simple serial measurement of the lateral ventricles. Percentile graphs for ventricular size have been published decades ago and were recently reviewed.^98–100^ Ventricular Index (VI) and anterior horn width (AHW), both obtained on a coronal plane passing through the foramina of Monro, are commonly used parameters. However, the absence of significant widening of the frontal horns may sometimes lead to under-estimation of PHVD severity, as neonates tend toward colpocephaly.^101^ Thalamo-occipital distance (TOD) is measured on the parasagittal plane and reflects the degree of dilatation of the trigone and the occipital horn of each lateral ventricle. Reproducibility of these ventricular measurements (VI, AHW and TOD) was at least good in two ultrasound studies assessing both intra- and inter-observer reliability.^99,100^ VI and AHW are used in most European NICUs to define the threshold for PHVD treatment: although no international consensus exists on optimal timing, the majority of European Centres initiate treatment once the ventricular width has crossed the 97th percentile + 4 mm line on Levene’s graph.^102^ Common findings in severe PHVD include a rounded upper border of the frontal horns on coronal planes (also referred to as “ballooning”) and a rounded anterior profile of the third ventricle in the midsagittal plane, due to the markedly dilated infundibular and supraoptic recess. Moreover, a dilated fourth ventricle can be identified in the midsagittal plane in case of tetraventricular or even communicating hydrocephalus. Besides these basics, the routine use of additional acoustic windows can provide useful information to understand the type of PHVD the patient has developed: insonation above the ear, can show a markedly dilated aqueduct in tetraventricular or communicating hydrocephalus, as well as an obstructed aqueduct in triventricular hydrocephalus.^75^ By gently tilting and rotating the probe on a pseudo-axial plane, the sonographer should aim to depict, in the same image, third and fourth ventricle connected by the aqueduct (Fig. 8).

### Doppler findings and GMH-IVH

**Fig. 9:**
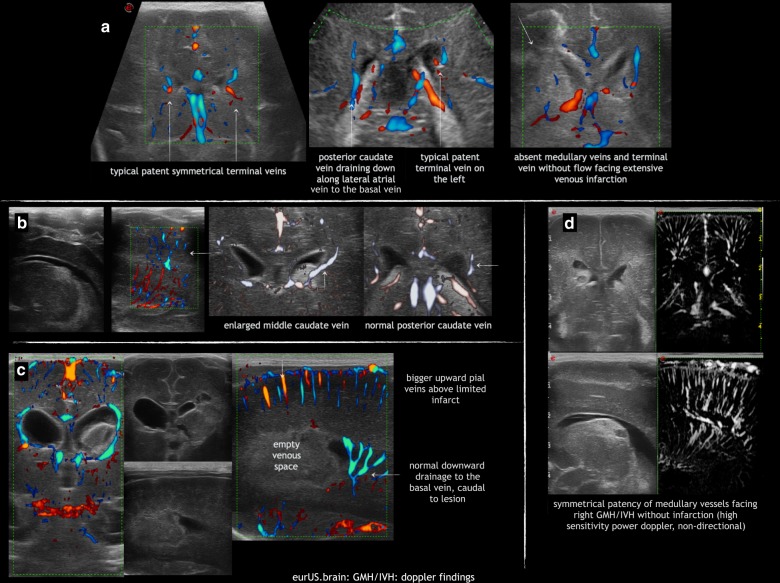
GMH/IVH: Doppler findings. **a** Asymmetry in terminal vein size, inversely related to size of ipsilateral direct lateral vein or lateral atrial vein; to be compared with absent terminal vein facing extensive venous infarction. **b** Apparent enlargement (congestion) of a vein by GMH. **c** Escape of blood from deep venous infarct along enlarged pial veins, above a Doppler-empty venous space. **d** Normal deep venous anatomy in GMH without infarction.

Although observations were made decades ago about visualization of deep veins in relation to GMH, this is an area where more research is needed. There are not enough Doppler findings published about flow changes near GMH-IVH. Improvement of the Doppler resolution does provide the opportunity to study the terminal vein and its tributaries^103,104^ (Fig. 9). Besides quality of technique (very different between vendors) an important pitfall in interpretation of Doppler observations is the high percentage of variation in deep vein anatomy. The septal and internal cerebral veins are near constantly present in viable preterm infants, but all other veins are not.^105^ Atrial veins and the direct lateral vein can be observed and their anatomy related to the presence of ipsilateral GMH. Variations in deep venous anatomy may either protect against infarction or on the contrary predispose to it; the extent of pial collateralisation may determine infarct size. Future studies may also focus on prevention of venous infarction when a large GMH is observed on admission.

Ultrafast Doppler, a recently developed ultrasound technique, allowed the introduction of functional CUS in animal models as well as in human neonates. Functional CUS detects changes in regional cerebral blood flow triggered by specific patterns of sensory stimulation, by different stages of sleep and by seizures. It has been estimated that this promising technique will be available for the introduction in the clinical setting within a few years.^106^

## Conclusion

State of the art ultrasound devices with high-frequency transducers allow today very detailed diagnosis of GMH-IVH/PHI with exact anatomical description of localization and extent of the lesion beyond the up to date commonly used grading systems. We encourage neonatologists and ultrasonographers to take advantage of the impressive progress in CUS imaging quality in order to improve disease description and classification in the routine clinical practice. In the near future, an accurate classification of the lesions combined with the knowledge deriving from the clinical application of modern ultrasound techniques (e.g. functional CUS through ultrafast Doppler) may lead to more precise prediction of outcome and open new horizons in the understanding of pathophysiology of brain injury and complications related to GMH-IVH.

## Supplementary information

Video
